# Isobutyl 3,5-dinitro­benzoate

**DOI:** 10.1107/S1600536809010381

**Published:** 2009-03-28

**Authors:** Pei Zou, Min-Hao Xie, Shi-Neng Luo, Ya-Ling Liu, Yong-Jun He

**Affiliations:** a Jiangsu Institute of Nuclear Medicine, Wuxi 214063, People’s Republic of China

## Abstract

In the structure of the title compound, C_11_H_12_N_2_O_6_, the mol­ecules are stacked along the *b* axis without any π–π inter­actions. The stacked columns are linked together by non-classical inter­molecular C—H⋯O inter­actions,. In the molecule, the nitro groups make dihedral angles of 9.4 (5) and 10.3 (5)° with the benzene ring.

## Related literature

For the properties and applications of dinitro­benzoate derivatives, see: Huang *et al.* (2004 ▶); Kagitani *et al.* (1984 ▶); Olive (1979 ▶). For the anti-creatinine effects of a series of 3,5-dinitro­benzoic acid esters, see: Yu & Yang (2002 ▶). For bond-length data, see: Allen *et al.* (1987 ▶).
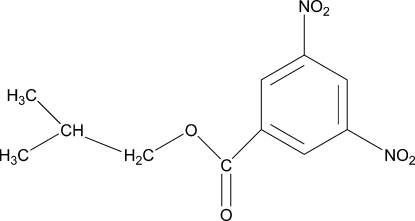


## Experimental

### 

#### Crystal data

C_11_H_12_N_2_O_6_
*M* *_r_* = 268.23
Monoclinic, 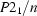

*a* = 16.666 (3) Å
*b* = 4.776 (1) Å
*c* = 16.678 (3) Å
β = 110.30 (3)°
*V* = 1245.1 (5) Å^3^
*Z* = 4
Mo *K*α radiation
μ = 0.12 mm^−1^
*T* = 293 K
0.30 × 0.20 × 0.10 mm

#### Data collection

Enraf–Nonius CAD-4 diffractometer
Absorption correction: ψ scan (North *et al.*, 1968 ▶) *T*_min_ = 0.965, *T*_max_ = 0.988
2348 measured reflections
2266 independent reflections
1402 reflections with *I* > 2σ(*I*)
*R*_int_ = 0.061
3 standard reflections every 200 reflections intensity decay: 1%

#### Refinement

*R*[*F* ^2^ > 2σ(*F* ^2^)] = 0.069
*wR*(*F* ^2^) = 0.225
*S* = 1.11
2266 reflections
172 parameters
H-atom parameters constrained
Δρ_max_ = 0.25 e Å^−3^
Δρ_min_ = −0.30 e Å^−3^

### 

Data collection: *CAD-4 Software* (Enraf–Nonius, 1989 ▶); cell refinement: *CAD-4 Software*; data reduction: *XCAD4* (Harms & Wocadlo,1995 ▶); program(s) used to solve structure: *SHELXS97* (Sheldrick, 2008 ▶); program(s) used to refine structure: *SHELXL97* (Sheldrick, 2008 ▶); molecular graphics: *SHELXTL* (Sheldrick, 2008 ▶); software used to prepare material for publication: *SHELXL97*.

## Supplementary Material

Crystal structure: contains datablocks I, global. DOI: 10.1107/S1600536809010381/pv2148sup1.cif

Structure factors: contains datablocks I. DOI: 10.1107/S1600536809010381/pv2148Isup2.hkl

Additional supplementary materials:  crystallographic information; 3D view; checkCIF report

## Figures and Tables

**Table 1:** Hydrogen-bond geometry (Å, °)

*D*—H⋯*A*	*D*—H	H⋯*A*	*D*⋯*A*	*D*—H⋯*A*
C7—H7*A*⋯O2^i^	0.93	2.52	3.441 (5)	168

Symmetry code: (i) 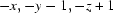.

**Fig. 1.**
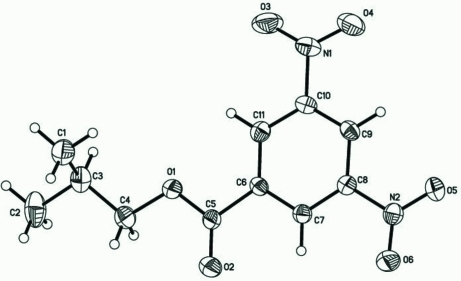
A view of the title compound with the atomic numbering scheme. Displacement ellipsoids were drawn at the 30% probability level.

## supplementary crystallographic information

### Comment

Due to their biological activities, dinitrobenzoate derivatives are widely used in pharmacology. Dinitrobenzoic acid derivatives are effective in tumour treatment as radiation sensitizers (Kagitani *et al.*, 1984). Moreover, some synthetic dinitrobenzoate compounds have shown useful properties in DNA and oligosaccharide synthesis (Olive, 1979; Huang *et al.*, 2004). Furthermore, a series of 3,5-dinitrobenzoic acid esters has also been synthesized and their anti-creatinine effects have been studied (Yu & Yang, 2002). To study their structures and activities, we report here the crystal structure of the title compound, (I).

The bond lengths and angles in (I) (Fig. 1) are within expected ranges (Allen *et al.*, 1987). The two nitro groups are inclined by 9.4 (5) and 169.7 (5)° to the benzene ring, respectively. Except for atoms C1 and C2, the other non-H atoms of the molecule lie in a plane. In the crystal structure, the molecules are stacked along the *b* axis, without any π-π interaction. The stacked columns are linked together by non-classical intermolecular interactions of the type C—H···O, details have been given in Table 1.

### Experimental

3,5-Dinitrobenzoylchloride (5200 mg, 23 mmol) was added in iso-butanol (25 ml, 271 mmol) and the mixture was refluxed for 4 h. White product appeared after cooling to room temperature. They were separated and washed with cold water. Single crystals of the title compound were grown by slow evaporation of a methanol solution: colourless needle-shaped crystals were formed after several days.

### Refinement

Positional parameters of all the H atoms bonded to C atoms were calculated geometrically and were allowed to ride on the C atoms to which they are bonded, with C—H distances of 0.93 Å(aromatic), 0.98 Å(CH), 0.97 Å(CH_2_) and 0.96 Å(CH_3_); *U*_iso_(H) = 1.2*U*_eq_(C) for the aromatic H, CH and CH_2_; *U*_iso_(H) = 1.5*U*_eq_(C) for CH_3_.

### Crystal data

**Table:**

C_11_H_12_N_2_O_6_	*F*(000) = 560
*M**_r_* = 268.23	*D*_x_ = 1.431 Mg m^−^^3^
Monoclinic, *P*2_1_/*n*	Mo *K*α radiation, λ = 0.71073 Å
Hall symbol: -P 2yn	Cell parameters from 25 reflections
*a* = 16.666 (3) Å	θ = 9–12°
*b* = 4.776 (1) Å	µ = 0.12 mm^−^^1^
*c* = 16.678 (3) Å	*T* = 293 K
β = 110.30 (3)°	Needle, colourless
*V* = 1245.1 (5) Å^3^	0.30 × 0.20 × 0.10 mm
*Z* = 4	

### Data collection

**Table:**

Enraf–Nonius CAD-4 diffractometer	1402 reflections with *I* > 2σ(*I*)
Radiation source: fine-focus sealed tube	*R*_int_ = 0.061
graphite	θ_max_ = 25.3°, θ_min_ = 1.5°
ω/2θ scans	*h* = 0→20
Absorption correction: ψ scan (North *et al.*, 1968)	*k* = 0→5
*T*_min_ = 0.965, *T*_max_ = 0.988	*l* = −20→18
2348 measured reflections	3 standard reflections every 200 reflections
2266 independent reflections	intensity decay: 1%

### Refinement

**Table:**

Refinement on *F*^2^	Primary atom site location: structure-invariant direct methods
Least-squares matrix: full	Secondary atom site location: difference Fourier map
*R*[*F*^2^ > 2σ(*F*^2^)] = 0.069	Hydrogen site location: inferred from neighbouring sites
*wR*(*F*^2^) = 0.225	H-atom parameters constrained
*S* = 1.11	*w* = 1/[σ^2^(*F*_o_^2^) + (0.1*P*)^2^ + *P*] where *P* = (*F*_o_^2^ + 2*F*_c_^2^)/3
2266 reflections	(Δ/σ)_max_ < 0.001
172 parameters	Δρ_max_ = 0.25 e Å^−^^3^
0 restraints	Δρ_min_ = −0.30 e Å^−^^3^

### Special details

**Table:**

Geometry. All e.s.d.'s (except the e.s.d. in the dihedral angle between two l.s. planes) are estimated using the full covariance matrix. The cell e.s.d.'s are taken into account individually in the estimation of e.s.d.'s in distances, angles and torsion angles; correlations between e.s.d.'s in cell parameters are only used when they are defined by crystal symmetry. An approximate (isotropic) treatment of cell e.s.d.'s is used for estimating e.s.d.'s involving l.s. planes.
Refinement. Refinement of *F*^2^ against ALL reflections. The weighted *R*-factor *wR* and goodness of fit *S* are based on *F*^2^, conventional *R*-factors *R* are based on *F*, with *F* set to zero for negative *F*^2^. The threshold expression of *F*^2^ > σ(*F*^2^) is used only for calculating *R*-factors(gt) *etc*. and is not relevant to the choice of reflections for refinement. *R*-factors based on *F*^2^ are statistically about twice as large as those based on *F*, and *R*- factors based on ALL data will be even larger.

### Fractional atomic coordinates and isotropic or equivalent isotropic displacement parameters (Å^2^)

**Table:**

	*x*	*y*	*z*	*U*_iso_*/*U*_eq_	
O1	−0.17180 (16)	−0.2811 (6)	0.58690 (17)	0.0567 (8)	
O2	−0.08913 (17)	−0.4218 (7)	0.51387 (18)	0.0616 (8)	
O3	−0.0648 (2)	0.3897 (9)	0.8166 (2)	0.0933 (13)	
O4	0.0522 (2)	0.6102 (7)	0.8340 (2)	0.0767 (10)	
O5	0.23983 (17)	0.2979 (6)	0.68814 (18)	0.0603 (8)	
O6	0.20462 (18)	−0.0604 (6)	0.60527 (19)	0.0637 (9)	
N1	−0.0004 (2)	0.4291 (8)	0.8000 (2)	0.0542 (9)	
N2	0.18975 (19)	0.1116 (7)	0.6515 (2)	0.0463 (8)	
C1	−0.2813 (3)	−0.6160 (11)	0.6546 (3)	0.0745 (14)	
H1A	−0.2346	−0.5124	0.6936	0.112*	
H1B	−0.2629	−0.8031	0.6491	0.112*	
H1C	−0.3278	−0.6224	0.6761	0.112*	
C2	−0.3856 (3)	−0.6279 (12)	0.5060 (3)	0.0864 (17)	
H2A	−0.4308	−0.6444	0.5289	0.130*	
H2B	−0.3680	−0.8112	0.4951	0.130*	
H2C	−0.4055	−0.5237	0.4535	0.130*	
C3	−0.3102 (3)	−0.4769 (9)	0.5697 (3)	0.0593 (11)	
H3A	−0.3289	−0.2870	0.5771	0.071*	
C4	−0.2420 (3)	−0.4536 (10)	0.5323 (3)	0.0645 (12)	
H4A	−0.2208	−0.6386	0.5264	0.077*	
H4B	−0.2651	−0.3702	0.4759	0.077*	
C5	−0.1000 (2)	−0.2845 (8)	0.5687 (2)	0.0418 (8)	
C6	−0.0337 (2)	−0.0901 (7)	0.6262 (2)	0.0363 (8)	
C7	0.0447 (2)	−0.0816 (7)	0.6143 (2)	0.0388 (8)	
H7A	0.0553	−0.1948	0.5736	0.047*	
C8	0.1063 (2)	0.0988 (7)	0.6641 (2)	0.0381 (8)	
C9	0.0934 (2)	0.2683 (7)	0.7252 (2)	0.0406 (8)	
H9A	0.1356	0.3901	0.7578	0.049*	
C10	0.0157 (2)	0.2503 (7)	0.7359 (2)	0.0407 (8)	
C11	−0.0482 (2)	0.0730 (8)	0.6876 (2)	0.0430 (9)	
H11A	−0.1002	0.0641	0.6965	0.052*	

### Atomic displacement parameters (Å^2^)

**Table:**

	*U*^11^	*U*^22^	*U*^33^	*U*^12^	*U*^13^	*U*^23^
O1	0.0495 (15)	0.0679 (19)	0.0601 (17)	−0.0169 (14)	0.0283 (13)	−0.0232 (14)
O2	0.0559 (17)	0.070 (2)	0.0611 (17)	−0.0124 (15)	0.0226 (14)	−0.0231 (16)
O3	0.072 (2)	0.123 (3)	0.096 (3)	0.002 (2)	0.043 (2)	−0.047 (2)
O4	0.093 (2)	0.066 (2)	0.070 (2)	−0.0068 (19)	0.0260 (18)	−0.0222 (18)
O5	0.0550 (16)	0.0615 (19)	0.0638 (18)	−0.0203 (15)	0.0200 (14)	−0.0124 (15)
O6	0.0572 (17)	0.067 (2)	0.075 (2)	−0.0002 (15)	0.0328 (15)	−0.0174 (17)
N1	0.056 (2)	0.053 (2)	0.0485 (19)	0.0094 (18)	0.0128 (16)	−0.0119 (17)
N2	0.0420 (17)	0.048 (2)	0.0478 (18)	0.0002 (15)	0.0143 (14)	0.0031 (16)
C1	0.073 (3)	0.085 (4)	0.069 (3)	−0.020 (3)	0.029 (2)	−0.008 (3)
C2	0.051 (3)	0.100 (4)	0.090 (4)	−0.023 (3)	0.001 (2)	0.023 (3)
C3	0.052 (2)	0.054 (3)	0.073 (3)	−0.004 (2)	0.022 (2)	0.006 (2)
C4	0.056 (2)	0.081 (3)	0.057 (2)	−0.024 (2)	0.020 (2)	−0.020 (2)
C5	0.048 (2)	0.040 (2)	0.0394 (19)	−0.0065 (17)	0.0181 (16)	0.0011 (17)
C6	0.0414 (18)	0.0324 (18)	0.0320 (17)	−0.0024 (15)	0.0088 (14)	−0.0001 (15)
C7	0.0446 (19)	0.0347 (18)	0.0377 (18)	0.0001 (16)	0.0151 (15)	0.0015 (15)
C8	0.0384 (18)	0.0371 (19)	0.0378 (18)	0.0030 (15)	0.0118 (15)	0.0040 (15)
C9	0.047 (2)	0.0325 (19)	0.0378 (18)	−0.0016 (16)	0.0091 (15)	−0.0001 (15)
C10	0.0456 (19)	0.038 (2)	0.0352 (18)	0.0048 (16)	0.0095 (15)	−0.0036 (15)
C11	0.0422 (19)	0.048 (2)	0.0387 (18)	0.0011 (17)	0.0134 (16)	0.0060 (17)

### Geometric parameters (Å, °)

**Table:**

O1—C5	1.332 (4)	C2—H2C	0.9600
O1—C4	1.462 (5)	C3—C4	1.479 (5)
O2—C5	1.190 (4)	C3—H3A	0.9800
O3—N1	1.213 (4)	C4—H4A	0.9700
O4—N1	1.222 (4)	C4—H4B	0.9700
O5—N2	1.228 (4)	C5—C6	1.505 (5)
O6—N2	1.210 (4)	C6—C11	1.373 (5)
N1—C10	1.463 (5)	C6—C7	1.390 (5)
N2—C8	1.478 (4)	C7—C8	1.377 (5)
C1—C3	1.485 (6)	C7—H7A	0.9300
C1—H1A	0.9600	C8—C9	1.375 (5)
C1—H1B	0.9600	C9—C10	1.369 (5)
C1—H1C	0.9600	C9—H9A	0.9300
C2—C3	1.517 (6)	C10—C11	1.381 (5)
C2—H2A	0.9600	C11—H11A	0.9300
C2—H2B	0.9600		
			
C5—O1—C4	116.0 (3)	O1—C4—H4A	109.6
O3—N1—O4	123.5 (4)	C3—C4—H4A	109.6
O3—N1—C10	118.5 (4)	O1—C4—H4B	109.6
O4—N1—C10	117.9 (3)	C3—C4—H4B	109.6
O6—N2—O5	123.7 (3)	H4A—C4—H4B	108.1
O6—N2—C8	118.3 (3)	O2—C5—O1	124.8 (3)
O5—N2—C8	118.0 (3)	O2—C5—C6	123.7 (3)
C3—C1—H1A	109.5	O1—C5—C6	111.5 (3)
C3—C1—H1B	109.5	C11—C6—C7	120.5 (3)
H1A—C1—H1B	109.5	C11—C6—C5	123.0 (3)
C3—C1—H1C	109.5	C7—C6—C5	116.5 (3)
H1A—C1—H1C	109.5	C8—C7—C6	118.2 (3)
H1B—C1—H1C	109.5	C8—C7—H7A	120.9
C3—C2—H2A	109.5	C6—C7—H7A	120.9
C3—C2—H2B	109.5	C7—C8—C9	122.7 (3)
H2A—C2—H2B	109.5	C7—C8—N2	118.8 (3)
C3—C2—H2C	109.5	C9—C8—N2	118.6 (3)
H2A—C2—H2C	109.5	C10—C9—C8	117.3 (3)
H2B—C2—H2C	109.5	C10—C9—H9A	121.4
C4—C3—C1	113.2 (4)	C8—C9—H9A	121.4
C4—C3—C2	108.1 (4)	C9—C10—C11	122.3 (3)
C1—C3—C2	111.8 (4)	C9—C10—N1	118.7 (3)
C4—C3—H3A	107.9	C11—C10—N1	119.0 (3)
C1—C3—H3A	107.9	C6—C11—C10	118.9 (3)
C2—C3—H3A	107.9	C6—C11—H11A	120.5
O1—C4—C3	110.2 (3)	C10—C11—H11A	120.5
			
C5—O1—C4—C3	168.4 (4)	O6—N2—C8—C9	171.3 (3)
C1—C3—C4—O1	−62.2 (5)	O5—N2—C8—C9	−9.2 (5)
C2—C3—C4—O1	173.4 (4)	C7—C8—C9—C10	0.6 (5)
C4—O1—C5—O2	−2.2 (6)	N2—C8—C9—C10	−179.5 (3)
C4—O1—C5—C6	177.4 (3)	C8—C9—C10—C11	−0.6 (5)
O2—C5—C6—C11	178.1 (4)	C8—C9—C10—N1	−179.6 (3)
O1—C5—C6—C11	−1.5 (5)	O3—N1—C10—C9	−171.3 (4)
O2—C5—C6—C7	−1.8 (5)	O4—N1—C10—C9	8.5 (5)
O1—C5—C6—C7	178.6 (3)	O3—N1—C10—C11	9.7 (5)
C11—C6—C7—C8	−1.5 (5)	O4—N1—C10—C11	−170.5 (3)
C5—C6—C7—C8	178.4 (3)	C7—C6—C11—C10	1.5 (5)
C6—C7—C8—C9	0.4 (5)	C5—C6—C11—C10	−178.4 (3)
C6—C7—C8—N2	−179.5 (3)	C9—C10—C11—C6	−0.4 (5)
O6—N2—C8—C7	−8.8 (5)	N1—C10—C11—C6	178.5 (3)
O5—N2—C8—C7	170.7 (3)		

### Hydrogen-bond geometry (Å, °)

**Table:**

*D*—H···*A*	*D*—H	H···*A*	*D*···*A*	*D*—H···*A*
C7—H7A···O2^i^	0.93	2.52	3.441 (5)	168

Symmetry codes: (i) −*x*, −*y*−1, −*z*+1.
